# Annual Meetings of Hospitals and Societies

**Published:** 1921-03-19

**Authors:** 


### Annual Meetings of Hospitals and Societies.

### The Power of the Pence.

The annual meeting of the Leicester and County Saturday Hospital Society was held at the Leicester Royal Infirmary on March 12. Mr. S. A. Gimson presided, and was supported by the Mayor of Leicester (Mr. G. A. Hilton), Mr. T. Fielding Johnson, jun., and Colonel C. J. Bond (Chairman and Deputy Chairman of the Infirmary). The delegates numbered nearly 300. The report shows a 50 per cent. increase in contributions on the previous year, being £32,666, which is a record. The income in 1919 was £21,631. The increased contribution is mostly accounted for by the new scale of weekly payments —twopence per week instead of one penny. In county areas the Fund works in active co-operation with local cottage hospitals. The latest branch of the Society's work—a convalescent home for children—the generous gift of Sir Arthur Wheeler, Bart., has been conspicuously successful. In the three homes (for men, women, and children respectively) upwards of 600 members and their dependants have received convalescent treatment, while 250 patients have been sent to special and other homes. The Society has been unfortunately handicapped during the year by the ill-health of the Organising Secretary, Mr. H. H. Woolley, J.P., in whose absence the control has passed to Mr. Abell, the Assistant Secretary. The total cost of maintenance of the Society was £12,798, £4,400 of which was largely on account of the alterations and equipment of the new children's home. The total contributions are divisible between the Infirmary (£19,760) and the Society (£11,760) after deduction of expenses.

### Samaritan Free Hospital for Women.

Dr. Oxford presided at the annual meeting of the Samaritan Free Hospital for Women on March 8. The report states that the total income for the year was £14,287, exceeding the expenditure by £2,445. In 1919 the income amounted to £9,244, and there was a deficit of £2,550. The improved financial position is attributable to the emergency grant of £1,500 from the King's Fund and the grant of £1,700 from the National Relief Fund. The loan amount has been reduced from £3,000 to £1,000. Voluntary weekly payments and donations from in-patients amounted to £1,072, and constituted a welcome addition to the income of the hospital. Very few patients have refused to contribute, and it is anticipated that the amount from this source will increase in the future. There has been a slight decrease in the cost of patients' maintenance per week—viz., from £3 5s. 11d. in 1919 to £3 4s. 2d. in 1920. The annual Pound Day realised £83 in cash and 672 lb. in goods, and was more successful than that of any previous year. The total deficit on the convalescent home of £734 has been met by grants from the General and Samaritan Funds. In recognition of the valuable surgical assistance he gave to the hospital during the war Dr. F. J. Booker was nominated as an Hon. Life Governor by the Committee.

The Chelsea Hospital for Women has received from the Goldsmiths' Company and the Grocers' Company £50 each towards building its Nurses' Home, and £12 18s. 9d., proceeds of an offertory from Chelsea Old Church.

